# High resolution chest computed tomography findings in patients with clinically suspected COVID-19 pneumonia in Uganda: a cross-sectional study

**DOI:** 10.4314/ahs.v23i4.12

**Published:** 2023-12

**Authors:** Rita Nassanga, Aloysius Gonzaga Mubuuke, Randhawa Mangun, Max Crescent Tumusiime, Erem Geoffrey, Valeria Nabbosa, Francis Olweny, Faith Ameda, Sam Bugeza

**Affiliations:** 1 Makerere University College of Health Sciences, Department of Radiology; 2 Massachusetts General Hospital, Department of Radiology; 3 St Francis Hospital, Nsambya, Department of Radiology; 4 Uganda Cancer Institute; 5 Makerere University College of Health Sciences, Department of Epidemiology and Biostatics

**Keywords:** CORADS, COVID-19, Chest HRCT

## Abstract

**Background:**

The alarming spread of the COVID-19 pandemic has led to a shortage of RT-PCR kits in Uganda necessitating the use of high-resolution chest Computed Tomography to guide patient management and treatment.

**Main Objective:**

To describe the chest HRCT findings in patients with clinically suspected COVID-19 pneumonia and to compare its diagnostic accuracy to RT-PCR. .

**Methods:**

In this cross-sectional study, chest HRCT findings of 384 patients and available RT-PCR laboratory results were reviewed and recorded in the data collection form.

**Results:**

The commonest chest HRCT findings were bilateral ground glass opacities (78.2%). Out of the 31.7% patients that took the PCR test only 26.9% tested positive. 16 out of 17 patients who tested negative, were classified under CORADS 5.

The sensitivity of chest HRCT was 90.4%, 95% CI (82.6–95.5), positive predictive value of 84.2%, 95% CI (75.6–90.7), and accuracy of 77.5%, 95% CI (71.5–87.1).

**Conclusions:**

HRCT was found superior to RT-PCR in diagnosing COVID-19. A patient with positive HRCT findings should be treated as COVID 19 when RT-PCR is inaccessible or results are negative. A patient with negative HRCT requires complimentary RT-PCR and possibly follow up CT scans if symptoms persist before treating for COVID 19.

## Background

Coronavirus disease 2019 (COVID-19) is caused by severe acute respiratory syndrome coronavirus 2 (SARS-CoV-2) that primarily affects the lungs. SARS-CoV-2 is a highly contagious respiratory disease and its first outbreak was reported in Wuhan, China 1. On January 30, 2020 the World Health Organization (WHO) declared it a pandemic and has since been reported in every country in the world.

As of September 30, 2021, a total of 219,000,000 cases of COVID-19 and 4,550,000 deaths due to COVID-19 were reported worldwide. Uganda reported its first case of COVID-19 on March 21, 2020 and by November30th, 2022, Uganda had registered 170,000 cumulative COVID-19 cases and 3,630 COVID-19 related deaths. COVID-19 causes severe respiratory distress due to primary involvement of the respiratory system; hence high-resolution chest computed tomography (HRCT) is highly recommended in clinically suspected COVID-19 cases in the initial evaluation of the disease, assessing disease progression and evaluating therapeutic efficacy 2, which shows results in accordance with the COVID-19 Reporting and Data System (CORADS), severity and staging of the disease. The CORADS is a standardization for reporting HRCTs in COVID-19 and it grades the findings on the likelihood of the diagnosis of COVID-19^2^. Currently, two severity scores are being used for HRCT; one scored out of 25 and another scored out of 40. The CT severity score out of 25 is graded per lobe as 0- No involvement, 1-Less than 5%, 2- 5-26% involvement, 3-25-50%, 4-51-75%, 5->75% with a global CT score being the sum of each lobe being maximum at 25. A score of 8 is mild, 9-15 is moderate and greater than 15 is severe 3. A score of greater than 18 is associated with high mortality.

The 40-point CT severity score is calculated by attributing a score of 0, 1 or 2 to each of the 20 regions in the lungs, if parenchymal opacification was none, less than 50%, or 50% or more, respectively. The overall CT severity score is a summation of scores of all 20 regions of both lungs combined, ranging from 0 to 40 points 3.

Recent studies addressing the importance of chest CT examination in COVID-19 patients, have reported CT sensitivity as 98% 4 however, the current reference standard for confirming the diagnosis of SARS-CoV-2, the Real-time reverse transcription–polymerase chain reaction (RT-PCR) on the nasal and pharyngeal swabs, only has a sensitivity of 32-80 % 5. In the absence of a reliable single diagnostic test for COVID-19, the role of chest CT became paramount in the diagnosis, evaluation and management of COVID-19. HRCT of the chest is a useful tool in the early diagnosis of COVID-19 infection, especially in settings of limited availability of RT-PCR like Uganda.

Furthermore, there is a general lack of testing kits in resource-poor settings. Against the backdrop of the urgent need for diagnosis given the high infectivity of the virus and the shortage of RT-PCR, HRCT has been used to diagnose COVID-19 pneumonia, with good sensitivity 6. Being novel pneumonia, there is still a paucity of data from sub-Saharan Africa which has also had a smaller number of COVID-19 patients compared to the rest of the world.

There is no published literature from Uganda regarding chest HRCT characteristics of patients with COVID-19 . The purpose of this study, therefore, was to describe the chest HRCT findings (severity score, stage of disease, and CORADS classification) in patients with clinically suspected COVID-19 pneumonia and to establish its diagnostic accuracy.

## Methods

### Design and setting

This was a cross-sectional descriptive retrospective study. The study was conducted in the Radiology Department of St Francis Hospital Nsambya which is a private not-for-profit (PNFP) hospital. This 500-bed faith-based hospital was chosen because of its location within the city and the large volume of patients it receives, being a major treatment centre for COVID-19 in the country.

### Study population

The patient population of this study consisted of adults with suspected COVID-19 pneumonia who were referred for a Chest CT scan between March 2020 and October 2021. A sample size of 384 participants whose HRCT images were reviewed was calculated using the Kish and Leslie formula. A sample size of 265 was estimated for diagnostic accuracy using a formula for estimating sample size for diagnostic test by [Bibr R7][Bibr R7].

In the formula we considered prevalence of COVID 19 to be 95% (COVID-19 Results Briefing Uganda December 15, 2022) and specificity to be 80% 8. Z-value corresponding to 95% confidence of 1.98 and maximum marginal error of estimate (d) of 0.05 were also considered. Consecutive sampling for the HRCT images was used.

### Inclusion criteria

Adequately filled request forms of all adult patients with symptoms of clinical COVID-19 pneumonia who were referred to the Radiology Department for chest HRCT, available chest HRCT scan images and reports were included.

### Exclusion criteria

Incomplete request forms and suboptimal chest HRCT scans were excluded. The suboptimal chest HRCT images were those that had significant respiratory artefacts and poor inspiration that markedly reduced the diagnostic quality.

### Study procedures

Chest HRCT scans were performed using a single inspiratory phase in a SIEMENS 128 slice CT scanner where the tube voltage was 130kVp with automatic tube current modulation. The CT images were reconstructed as axial images with a matrix size of 512 ×512 and a slice thickness of 1.0mm.

A structured data collection tool was used to collect information on study variables from patient records including: socio-demographic characteristics, clinical history characteristics, vaccination history, physical examination findings, oxygen saturation, RT-PCR test results, HRCT findings including severity score, stage of disease and CORADS classification.

The HRCT scan findings were reviewed and the reported findings were recorded in the data collection form. For missing chest HRCT reports, a review of the patient images was done by the study PI and six study radiologists. Image analysis was performed using the institutional digital database system by two radiologists, RN and BS who respectively have seven- and twelve-years experience of reporting chest HRCT scans. The radiologists who reported on the HRCT images were not blinded to patients' clinical history however they were blinded to RT-PCR information.

The independent variables were socio-demographic features, clinical features, and physical examination features whereas the dependent variables were HRCT report findings. The HRCT findings were 1) CT severity score categorized as normal, mild, moderate and severe, 2) disease stage categorized as stage 1 to 4 and 3) CORADS classification scored as CORADS 0 to 5.

### Data management and analysis

The collected data in the data extraction forms was double entered by two different research assistants into Epidata entry client V4.6.0.4 which was later exported through Epidata manager V4.6.0.4 to STATA statistical software V14.0 for cleaning and analysis. Categorical variables were summarized using frequencies and percentages. Comparisons between categorical variables was achieved using Pearson's Chi square test. Fisher's exact test was also used for comparisons in circumstances were assumptions for using Chi square test was violated. Statistical significance was considered at p<0.05.

To obtain diagnostic values, binary result of RT-PCR (negative and positive categories) was tabulated against binary categories of CORADS classification. CORADS classification scores/categories of 0-4 were considered negative whereas CORADS score 5 was considered positive. Diagnostic test was then run in STATA V14 using the command DIAGT 9 to obtain diagnostic values (sensitivity, specificity, positive and negative predictive values) and their corresponding 95% confidence intervals.

### Quality control

The data collection tool was pre-tested before commencing the study to ascertain if the required information could be obtained using the specific questions. The research assistants were trained in data collection procedures. Collected data was cross-checked at the end of every day for completeness. The data collection tools were stored in a secure place only accessible to the research team.

### Ethical consideration

Ethical approval to conduct the study was granted by the Nsambya Hospital Research Ethics Committee (REC No SFHN-2021-31:). Waiver of informed consent was also obtained since we were reviewing images retrospectively. All information collected was treated with confidentiality.

## Results

### Flow chart

**Figure 1:**
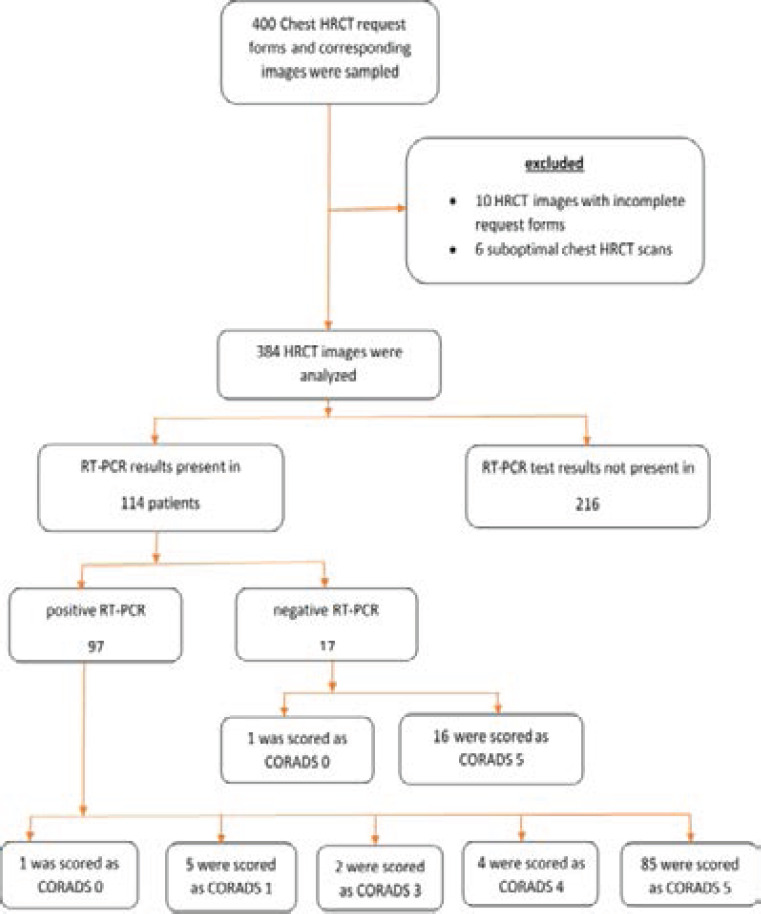
Flow chart

We sampled 400 HRCT images and their corresponding request forms. Of these, 10 HRCT images were excluded due to incomplete request forms and 6 were excluded due to suboptimal chest HRCT scans. We therefore analysed 384 HRCT images of suspected COVID-19 patients. This is summarized in figure 1 above.

### Socio-demographic characteristics

There were 384 participants. The mean age of participants in this study was 52 years (SD-17.1). The majority of these patients were aged 41-60 years (n=137, 39.0%), followed by 18-40 years (n=98, 28.0%). The male patients were more than females, 191(54.7%).

### Symptoms and corresponding duration

Most patients reported to have had at least two symptoms 146 (40.7%), of which cough 203 (63.0%) with an average duration of 8.7 days (SD, 4.7 days) was the most frequently reported symptom followed by difficulty in breathing at 87 (31.5%) with an average duration of 6.8 days (SD, 3.5 days) and fever 52 (18.8) with an average duration of 8.0 days (SD, 4.8 days). (Fig. 1)

**Figure 1:**
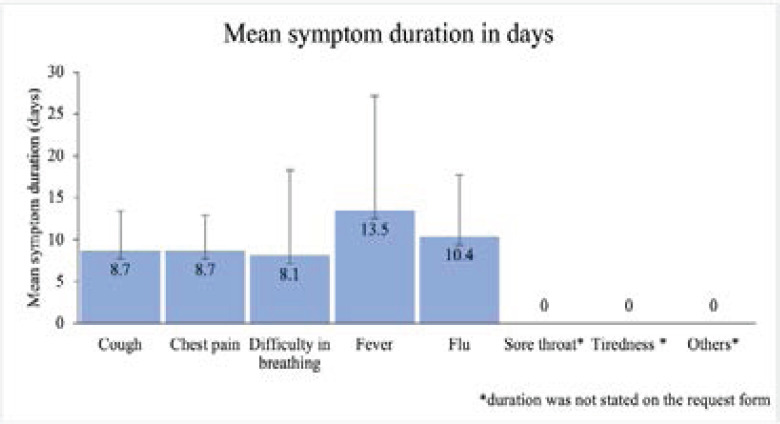
Symptoms and corresponding duration reported by suspected COVID-19 patients undergoing chest HRCT evaluation

### Chest HRCT findings

**Figure 3:**
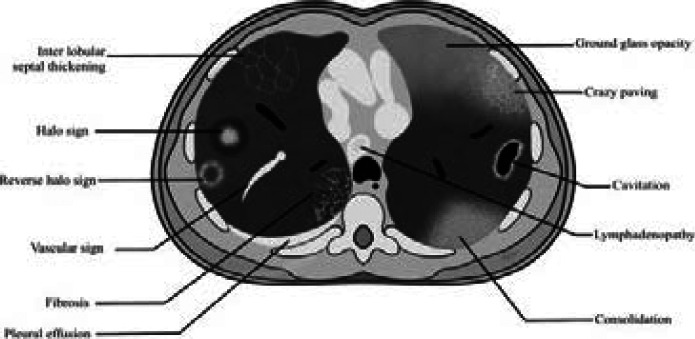
A diagrammatic representation of the pathological findings of COVID-19 infection as seen on HRCT imaging

Figure 3 provides a diagrammatic representation of the pathological findings of COVID-19 infection as seen on HRCT imaging.

**Figure 2:**
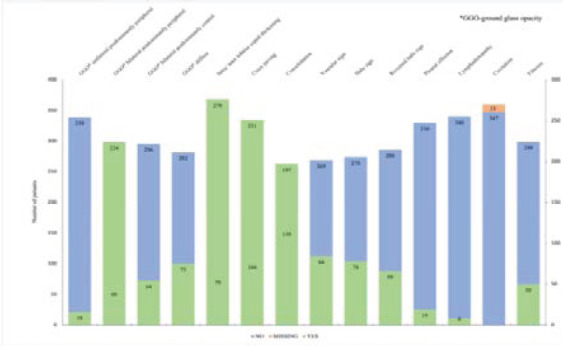
HRCT chest features of patients with suspected COVID-19 pneumonia. For each of the HRCT findings (labeled above the bars) : Blue = negative result; Green = positive result; Orange = missing result information

**Figure 4:**
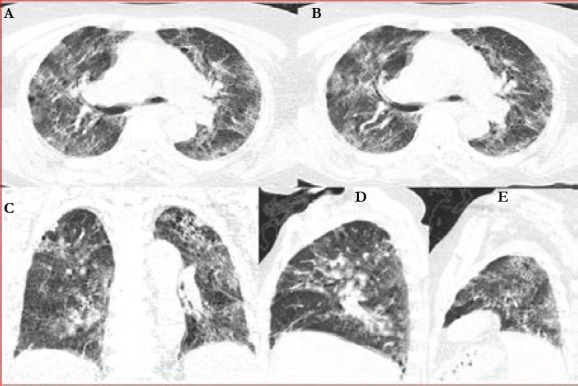
Axial (A, B), coronal(C), and sagittal (D, E) chest HRCT-lung windows of a 51-year-old male showing ground glass opacities with interlobular septal thickening and crazy paving pattern. This patient was categorised as CORADS 5, stage 3 with a severity score of 21/25

**Figure 5:**
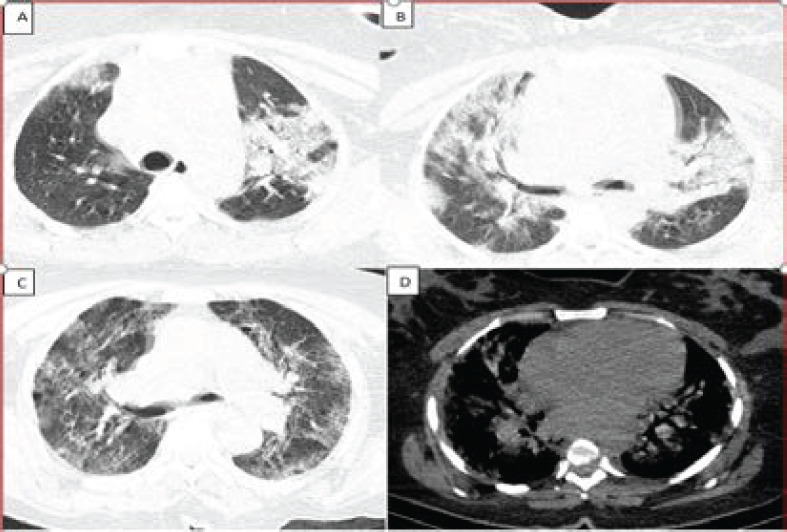
Axial lung (A, B, C) and mediastinal(D) chest HRCT windows of a 32-year-old female patient showing bilateral multilobar ground glass opacities and airspace disease with a peripheral predominance, as well as diffuse interlobular septal thickening. This patient was categorized as CORADS 5, stage 3 with a severity score of 17/25

**Figure 7:**
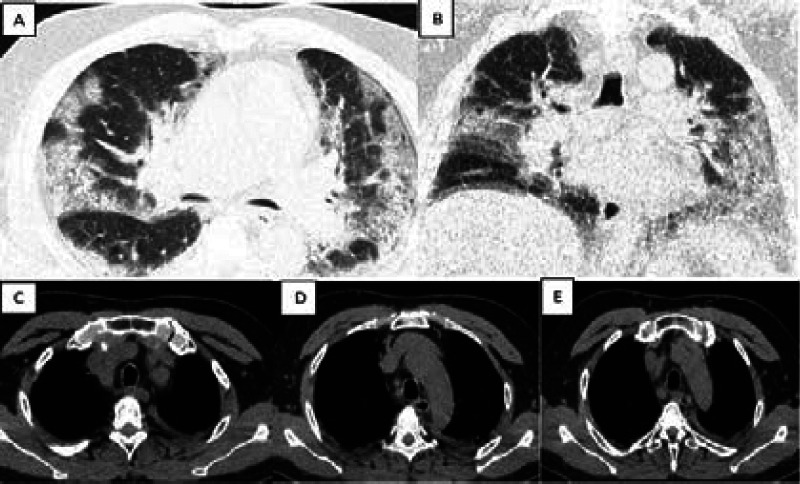
67-year-old with Diabetes and Hypertension, negative RT PCR who presented with difficulty in breathing for 5 days. Axial (, A, B) Chest HRCT shows diffuse GOO with a peripheral predominance. Axial Chest CT scans (C, D, E) show superior mediastinal lymphnodes. He was classified as CORADS 5, stage 3, with a severity score of 20/25

The most dominant features on chest HRCT of suspected COVID-19 patients undergoing evaluation were: bilateral ground glass opacities with peripheral predominance 224(78.2%) (Fig 4, 5, and 7), intra-lobular/inter-lobular septal thickening 278(77.8%), and crazy paving 251(70.7%) (Fig 4). Cavitation as a chest HRCT feature was not present in any of the suspected Covid-19 patients under evaluation (Fig. 2).

Severe disease 143 (41.6%) was the most observed category of severity score noted on the chest HRCT (Table 1). Furthermore, stage 3 as a category of disease staging was the most frequently observed 193(56.1%) (Fig 4, 5 and 7) and the majority of suspected COVID-19 patients were categorized under CORADS five ((Fig 4, 5 and 7).

**Table 1:** Chest HRCT severity score, stage and CORADS score of suspected Covid-19 patients who underwent radiological evaluation at St Francis Hospital Nsambya between 21^st^ March 2020 – 30^th^ September 2021

	Overall	Male	Female	
HRCT image findings	n (%)	n (%)	n (%)	p-value
**Severity score**				
Normal	8 (2.3)	4 (2.1)	4 (2.7)	
Mild	67(19.5)	37(19.5)	30(19.9)	
Moderate	126(36.6)	69(36.3)	55(36.4)	
Severe	143 (41.6)	80 (42.1)	62 (41.1)	0.994
**Disease stage**				
Stage 1	25(7.3)	14(7.4)	11(7.2)	
Stage 2	77(22.4)	41(21.6)	35(23.0)	
Stage 3	193(56.1)	110(57.9)	83(54.6)	
Stage 4	49(14.2)	25(13.2)	23(15.1)	0.924
**CORADS**				
Zero	2(0.6)	2(1.0)	0(0.0)	
One	6(1.7)	2(1.0)	4(2.6)	
Two	2(0.6)	1(0.5)	1(0.6)	
Three	9(2.5)	5(2.6)	4(2.6)	
Four	11(3.1)	6(3.1)	5(3.2)	
Five	324(91.5)	178(91.8)	142(91.0)	0.797

### PCR test findings

Only 114 patients had their PCR results read by the time chest HRCT images were acquired. 26.9% of the patients had a positive PCR test and the majority (68.3%) had not taken the PCR test.

Of the 17 patients with a negative PCR test, 16 (94%) of them were classified under CORADS 5. Furthermore, among the 216 patients who did not have PCR results, 200 (92.6%) were classified under CORADS 5, 27 (12.9%) of them were classified under stage 4 and the majority were classified under stage 3, 116 (55.5%).

### Performance of chest HRCT compared to PCR

When compared to RT-PCR as the gold standard, the sensitivity of chest HRCT was 90.4%, (95%CI; 82.6-95.5) with a positive predictive value of 84.2%, (95%CI; 75.6-90.7). The specificity of the HRCT was 5.9% (95% CI; 0.1-28.7). The overall accuracy of HRCT was 77.5% (95%CI; 71.5-87.1). The positive and negative predictive values were 84.2% (95% CI; 75.6-90.7) and 10.0% (95% CI; 0.3-44.5)- respectively. (Table 2)

**Table 2:** Accuracy of chest HRCT compared to RT-PCR among suspected COVID-19 patients

Performance	Percentage	CI
Sensitivity	90.4	82.6 - 95.5
Specificity	5.9	0.1 - 28.7
PPV	84.2	75.6 - 90.7
NPV	10.0	0.3 - 44.5
Prevalence	84.7	76.6 - 90.8
Accuracy	77.5	71.5 - 87.1

### Comorbidities, oxygen saturation, and vaccination with CORADS classification

The main comorbidity identified among suspected COVID-19 patients was hypertension, 17 (26.6%) followed by diabetes mellitus, 9 (14.1%) (Table 3). The relationship between CORADS and comorbidities among suspected COVID-19 patients at Nsambya hospital was insignificant (p 0.75) (Table 3). It is shown however that patients who were diagnosed with both hypertension and diabetes mellitus and underwent HRCT evaluation were all categorized as CORADS-5, 19 (100.0%) (Table 3).

**Table 3:** Relationship between comorbidities, oxygen saturation and vaccination with CORADS among suspected Covid-19 patients who underwent chest HRCT evaluation at St Francis Hospital Nsambya between 21^st^ March 2020 — 30^th^ September 2021

Variable	n(%)	CORADS					P-value**
		0	1	3	4	5	

		n(%)	n(%)	n(%)	n(%)	n(%)	
**Comorbidities/ Conditions**							
DM	9(14.1)	*	*	0(0.0)	0(0.0)	9(100.0)	
HTN	17(26.6)	*	*	1(5.9)	1(5.9)	15(88.2)	
Gravid	3(4.7)	*	*	0(0.0)	0(0.0)	3(100.0)	
postpartum	1(1.6)	*	*	0(0.0)	0(0.0)	1(100.0)	
malignancy/metastases	5(7.8)	*	*	0(0.0)	0(0.0)	5(100.0)	
HTN+/CKD+/ESRD+/DM	4(6.3)	*	*	0(0.0)	0(0.0)	4(100.0)	
Aortic Aneurysm	1(1.6)	*	*	0(0.0)	0(0.0)	1(100.0)	
Obesity	2(3.1)	*	*	0(0.0)	0(0.0)	2(100.0)	
HTN+DM	19(29.7)	*	*	0(0.0)	0(0.0)	19(100.0)	
HTN+ISS	1(1.6)	*	*	0(0.0)	0(0.0)	1(100.0)	
HTN+ malignancy	1(1.6)	*	*	0(0.0)	0(0.0)	1(100.0)	
DM+ malignancy/metastases	1(1.6)	*	*		0(0.0)	1(100.0)	0.755
**Oxygen Saturation (SPO2)**		*	*				
Low	83(82.2)	*	*	1(1.2)	1(1.2)	80(97.6)	
Normal	18(17.8)	*	*	3(16.7)	1(5.6)	14(77.8)	**0.009**
**Received COVID-19 vaccine**							
Yes	12(6.4)	0(0.0)	0(0.0)	0(0.0)	0(0.0)	12(100.0)	
No	188(92.2)	1(0.5)	2(1.1)	5(2.7)	2(1.1)	176(94.6)	
Not known	3(1.5)	0(0.0)	0(0.0)	0(0.0)	0(0.0)	3(100.0)	1.000

* _*no response was captured in CORADS 0 and 1*, ** _*p-value corresponding to Fisher's exact test, DM-diabetes mellitus, HTN – hypertension, ISS – immunosuppression syndrome, CKD – chronic kidney disease, ESRD- End stage renal disease*.

**Figure 6:**
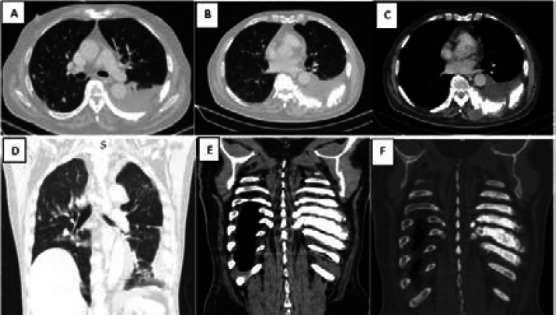
75-year-old male with confirmed Cancer of the prostate and RT PCR positive who came in with cough and difficulty in breathing; Axial (A, B, C) and coronal chest CT scans show scattered right lung nodules, fibrosis, scattered ground glass opacities and a left pleural effusion. Coronal (E, F) chest CT scans of mediastinal and bone window respectively, show irregularity, enlargement and sclerosis of the left 6th-10th posterior ribs in keeping with blastic metastases. The lung findings were suspicious for COVID-19 and were categorized as CORADS 4, stage 2 with a severity score of 10/25

The chest HRCT findings in a 75-year-old male who presented with cough and difficulty in breathing, with cancer of the prostate were lung metastases, blastid rib metastases and a left pleural effusion in addition to scattered ground glass opacities. The lung findings were suspicious for COVID-19 and were categorized as CORADS 4, stage 2 with a severity score of 10/25 (fig 6). Among suspected patients for COVID-19 in this study who had their SPO2 taken and recorded, the majority had low oxygen saturation (SPO2 < 95), 83 (82.2%). There was a significant relationship between oxygen saturation and CORADS classification, p 0.009 (Table 3). Most suspected patients for COVID-19 in this study 188 (92.2%) reported that they had not been vaccinated by the time HRCT was done. However, all patients who reported to have received vaccination for COVID-19 had chest HRCT CORADS classification of 5. This relationship was not significant, p 1.000 (Table 3).

### Comorbidities, oxygen saturation, and vaccination related to HRCT severity score

In-addition, we related the patient comorbidities, oxygen saturation and vaccination status to the HRCT severity score (Table 4). The relationship between HRCT severity score and comorbidities among suspected COVID-19 patients at Nsambya hospital was insignificant *p* 0.839). Patients who were diagnosed with both hypertension and diabetes mellitus and underwent HRCT evaluation were mostly scored as having severe disease, 10 (52.6%). The results also demonstrate that patients with severe disease under HRCT severity score of severe also had low oxygen saturation, 51 (63.0%). A significant association was noted between HRCT severity score and oxygen saturation, p < 0.001 (Table 4).

**Table 4:** Relationship between comorbidities, oxygen saturation and vaccination with HRCT severity score among suspected Covid-19 patients who underwent chest HRCT

Variable	n (%)	Severity score				P-value**
		Normal	Mild	Moderate	Severe	

		n (%)	n (%)	n (%)	n (%)	
**Comorbidities/ Conditions**						
DM	9(14.1)	0(0.0)	1(14.3)	4(57.1)	2(28.6)	
HTN	17(26.6)	1(5.9)	4(23.5)	4(23.5)	8(47.1)	
Gravid	3(4.7)	0(0.0)	0(0.0)	2(66.7)	1(33.3)	
Postpartum	1(1.6)	0(0.0)	1(100.0)	0(0.0)	0(0.0)	
Malignancy/metastases	5(7.8)	0(0.0)	0(0.0)	3(60.0)	2(40.0)	
HTN+/CKD+/ESRD+/DM	4(6.3)	0(0.0)	0(0.0)	2(50.0)	2(50.0)	
Aortic aneurysm	1(1.6)	0(0.0)	0(0.0)	0(0.0)	1(100.0)	
Obesity	2(3.1)	0(0.0)	1(50.0)	1(50.0)	0(0.0)	
HTN+DM	19(29.7)	0(0.0)	3(15.8)	6(31.6)	10(52.6)	
HTN+ISS	1(1.6)	0(0.0)	0(0.0)	0(0.0)	1(100.0)	
HTN+ malignancy	1(1.6)	0(0.0)	0(0.0)	0(0.0)	1(100.0)	
DM+ malignancy/metastases	1(1.6)	0(0.0)	0(0.0)	1(100.0)	0(0.0)	0.839
**Oxygen Saturation (SPO2)**						
Low	83(82.2)	0(0.0)	6(7.4)	24(29.6)	51(63.0)	
Normal	18(17.8)	1(5.6)	5(27.8)	10(55.6)	2(11.1)	<0.001
**Received COVID-19 vaccine**						
Yes	13(6.4)	1(10.0)	1(10.0)	6(60.0)	2(20.0)	
No	188(92.2)	2(1.1)	39(21.3)	64(35.0)	78(42.6)	
Not known	3(1.5)	0(0.0)	1(33.3)	1(33.3)	1(33.3)	0.153

** _*p-value corresponding to Fisher's exact test, DM-diabetes mellitus, HTN – hypertension, ISS – immunosuppression syndrome, CKD – chronic kidney disease, ESRD- End stage renal disease*.

The majority of suspected patients for COVID-19 who received vaccination were classified under an HRCT severity score of moderate, 6 (60.0%).

### Comorbidities, oxygen saturation, and vaccination related to HRCT staging

Lastly, we related patient comorbidities, oxygen saturation and vaccination status to the HRCT staging (Table 5). 58.8% (n=10) of the suspected patients for COVID-19 who were diagnosed with both hypertension and diabetes mellitus, were classified as stage 3 on HRCT. The association between comorbidities and HRCT staging was not significant, p 0.633 (Table 5).

**Table 5:** Relationship between comorbidities, oxygen saturation and vaccination with HRCT staging among suspected Covid-19 patients who underwent chest HRCT

Variable	n (%)	CT scan staging				P-value**
		Stage 1	Stage 2	Stage 3	Stage 4	

		n (%)	n (%)	n (%)	n (%)	
**Comorbidities/ Conditions**						
DM	9(14.1)	1(11.1)	3(33.3)	4(44.4)	1(11.1)	
HTN	17(26.6)	3(17.7)	5(29.4)	7(41.2)	2(11.8)	
Gravid	3(4.7)	0(0.0)	0(0.0)	3(100.0)	0(0.0)	
Postpartum	1(1.6)	0(0.0)	1(100.0)	0(0.0)	0(0.0)	
Malignancy/metastases	5(7.8)	0(0.0)	5(100.0)	0(0.0)	0(0.0)	
HTN+/CKD+/ESRD+/DM	4(6.3)	0(0.0)	2(66.7)	1(33.3)	0(0.0)	
Aortic aneurysm	1(1.6)	0(0.0)	0(0.0)	1(100.0)	0(0.0)	
Obesity	2(3.1)	0(0.0)	0(0.0)	2(100.0)	0(0.0)	
HTN+DM	19(29.7)	0(0.0)	4(23.5)	10(58.8)	3(17.7)	
HTN+ISS	1(1.6)	0(0.0)	0(0.0)	1(100.0)	0(0.0)	
HTN+ malignancy	1(1.6)	*	*	*	*	
DM+ malignancy/metastases	1(1.6)	0(0.0)	0(0.0)	0(0.0)	1(100.0)	0.633
**Oxygen Saturation (SPO2)**						
Low	83(82.2)	3(3.8)	16(20.3)	51(64.6)	9(11.4)	
Normal	18(17.8)	3(16.7)	4(22.2)	10(55.6)	1(5.6)	0.229
**Received COVID-19 vaccine**						
Yes	13(6.4)	1(8.3)	3(25.0)	6(50.0)	2(16.7)	
No	188(92.2)	17(9.4)	43(23.9)	95(52.8)	25(13.9)	
Not known	3(1.5)	0(0.0)	0(0.0)	3(100.0)	0(0.0)	0.959

* _*no response*, ** _*p-value corresponding to Fisher's exact test, DM-diabetes mellitus, HTN – hypertension, ISS – immunosuppression syndrome, CKD – chronic kidney disease, ESRD- End stage renal disease*.

64.6% (n=51) of the patients with low oxygen saturation demonstrated CT disease staging of 3. Oxygen saturation and HRCT disease staging were not significantly associated with each other, p 0.229.

What is the relationship between vaccination and HRCT staging?

## Discussion

The purpose of this study was to describe the chest HRCT scan findings in patients with COVID-19 and to compare the accuracy of HRCT with that of the current gold standard, PCR.^10^, ^11^, ^12^, ^13^, ^14^, ^15^, ^2^, ^11^,^3^,^3^, ^16^, ^17^

### Chest HRCT findings

The key findings included bilateral ground glass opacities (GGO) with peripheral predominance, intralobular/interlobular septal thickening, and crazy paving. These findings were comparable with what has been previously reported in literature 3, 15, 17-19. The reason for this similarity could be because of similar epidemiological factors that relate in these settings.

### Severity score

Currently, two severity scores are being used for HRCT; one scored out of 25 and another scored out of 40. The CT severity score out of 25 is graded per lobe as 0- No involvement, 1-Less than 5%, 2- 5-26% involvement, 3-25-50%, 4-51-75%, 5->75% with a global CT score being the sum of each lobe being maximum at 25. A score of 8 is mild, 9-15 is moderate and greater than 15 is severe 3. A score of greater than 18 is associated with high mortality. The 40-point CT severity score is calculated by attributing a score of 0, 1 or 2 to each of the 20 regions in the lungs, if parenchymal opacification was none, less than 50%, or 50% or more, respectively. The overall CT severity score is a summation of scores of all 20 regions of both lungs combined, ranging from 0 to 40 points 3.

In this study, we adopted the 25-point CT severity score and found a score of 15(severe) and above as the most observed category of severity score noted on the chest HRCT and this constituted 143 patients (41.6%) (Table 1). Comparing this to previous studies, one study reported a mean global CT score of moderate^20^ whereas in another study, 6.8% of patients 19% in ICU had severe disease with a score of more than 15 21. The reason for the severe disease in our setting could be related to factors such as self-medication and the belief that home remedies such as steaming and use of local herbs are helpful. Hence patients would report to the hospital after these preliminary attempts had failed.

Other studies using the 40-point CT-SS Severity score, 18.3% had severe disease 22 whereas the median HRCT severity score of patients with negative PCR results was more (15.5/40) than the patients with positive PCR (11/40) (13). A study of 103 patients by Sayeed et al, with a cut off of 19.5 out of 40 and above as being severe, 81% had severe disease 11, 23.

### HRCT stages

Temporal CT stages in COVID 19 involve; initial stage/stage 1-normal CT or GGO only, progressive stage/stage 2-increased GGO and crazy paving appearance, peak stage/stage 3-consolidation and absorption stage/stage 4- an improvement in the disease course, with fibrosis 3. In this study, the peak stage-stage 3 with a hallmark of consolidation was the most frequently observed. (Table 1). This happens at day 9-13 which is in line with our patients who presented at 6-10 days and relates well with previous literature that has reported similar findings 3 24, 25

### CORADS classification

COVID-19 Reporting and Data System (CO-RADS) is an initiative for standardization of reporting chest HRCT in patients suspected to have COVID-19. In CORADS 1 COVID-19 is highly unlikely, 2- Level of suspicion of COVID-19 infection is low, 3-COVID-19 is indeterminate, 4- CO-RADS 4 the level of suspicion is high where as in CORADS 5-the level of suspicion for COVID 19 is high 2, 26. Majority of suspected COVID-19 patients in this study were categorized under CORADS five (Table 1). In comparison to previous studies, our study had a higher CORADS score. 27. Conversely in our study, of the 17 patients who had negative RT-PCR test, 16of them were classified under CORADS 5. Therefore CORADS score has an upper hand as compared to CT severity score in predicting the severity and likelihood of COVID-19 as previously reported by Zayed et al 28.

In a study conducted by Bellini et al. to validate CO-RADS accuracy in diagnosing COVID-19 cases, the threshold value of 4 or more provided reasonable sensitivity and specificity of 61% and 81%, respectively 29. In another study, the same cutoff point has been used to predict severe COVID-19 cases and offered sensitivity and specificity about 88% and 98%, respectively 30. Our results were quite close to those of Prokop et al.'s 26, who found the accuracy of CO-RADS at 91%.

### Accuracy

In this study, chest HRCT had a sensitivity of 90.4%, positive predictive value of 84.2%, and accuracy of 77.5%These findings are comparable to previous studies 2, 26, 31,29, 30,26, 31.

Murtaza and colleagues found the sensitivity of typical HRCT to be 91.7-94.8% 31 in Pakistan while Shirin got a sensitivity, specificity, and accuracy of the CT scan diagnosis were found to be 83.2%, 50% and 79.9% in Bangladesh. A meta-analysis of 16 studies done in China and Japan showed that the HRCT chest is 86-96% sensitive in the diagnosis of COVID-19 pneumonia; in which the typical HRCT pattern for COVID 19 was defined as bilateral GGOs and multilobe consolidations 32. Another study found the specificity of HRCT in the detection of COVID-19 in CORADS IV and V to be very high (100%) 27. The false-negative rate for CO-RADS 1 was 5.6% and the false-positive rate for CO-RADS category 5 was 0.3%; 95% in the Netherlands 26.

As can be observed when compared with previous literature, our sensitivity was comparable however, our specificity and negative predictive value were very low at 5.9% and 10% respectively. The explanation for this is that the total number of patients who had results of both PCR and CORADS were only 111 and of the 111, some 85 were true positives, one true negative, 16 false positives, and 9 false negatives. Therefore, the low values for specificity and NPV were due to the one patient who was identified by both RT-PCR and chest HRCT as not having COVID-19.

### Comorbidities, oxygen saturation and vaccination in relation to CORADS, severity score, and staging

The main comorbidity identified among suspected COVID-19 patients was hypertension followed by diabetes mellitus and all the patients who were diagnosed with both hypertension and diabetes mellitus fell into the CORADS-5 category (Table 3). Majority of patients with both hypertension and diabetes mellitus were mostly scored as having severe disease. This finding is comparable to a study by Inanc and colleagues who reported that a high percentage of patients with hypertension and diabetes mellitus were scored as CORADS 5 33. Similar studies have also reported a strong association between a high CORADS score and comorbidities 4, 16, 34.

In addition, majority of patients with both hypertension and diabetes mellitus were scored as having severe disease and in peak stage 3. Human pathogenic coronaviruses such as SARS-CoV and SARS-CoV-2 use angiotensin-converting enzyme 2 (ACE2) receptors to bind to their target cells. ACE is expressed by epithelial cells of the lung, intestine, kidney, and blood vessels 35. The expression of ACE2 is higher in patients with diabetes and hypertension, who are treated with ACE inhibitors and angiotensin II type-I receptor blockers and thus the increased expression of ACE2 makes the lungs more vulnerable to injury and facilitates infection with COVID-19 with increased chances of respiratory failure 36. People with diabetes also have impaired phagocytosis and this makes them more prone to infections. Other mechanisms such as higher affinity cellular binding and efficient virus entry, decreased viral clearance, diminished T-cell function, increased susceptibility to hyperinflammation and cytokine storm syndrome may increase the susceptibility for COVID-19 in patients with diabetes mellitus 37.

Among the patients with low oxygen saturation (SPO2 < 95), majority scored CORADS 5 and oxygen saturation was significantly associated with CORADS classification. Patients who did not receive vaccination for COVID-19 had HRCT CORADS classification of 5 and this suggests that vaccination protects patients from getting severe disease. Majority of suspected patients for COVID-19 who received vaccination had moderate disease whereas 42.6% those who did not receive vaccination had severe disease. This is in agreement with previous literature that shows that non vaccinated patients are more likely to develop severe disease 38.

The strength of this study lies in its rigorous nature especially the use of multiple radiologists and scoring systems to interpret the CT findings which increased its reliability. Findings from the study do demonstrate the utility of HRCT that can be potentially useful in many settings when evaluating patients with suspected COVID-19 pneumonia and thus contribute to current debates that chest HRCT is a key contributor in assessing the severity, CORADS score and disease stage in COVID-19. A key limitation of the study was poor record keeping and some missing information. Achieving the required sample size was another hurdle due to reduced patient attendance in hospitals caused by the lockdown.

We thus recommend further research involving a larger sample size especially the use of retrospective reviews with the already existing COVID data in hospitals.

## Conclusion

In our setting, HRCT had a higher sensitivity in the diagnosis of COVID-19 compared to the gold standard RT-PCR. The RT-PCR test takes more time and requires readily available test kits, whereas HRCT scans require less time, is a non-invasive test and can provide results for immediate decision making for patient management. The majority of our patients had typical HRCT findings for COVID-19 and were treated as COVID-19 patients. . The chest HRCT is also fundamental in assessing the severity, CORADS score and disease stage in COVID-19 which helps with grading the disease and thus guides management. A patient with positive HRT findings should be treated as COVID-19 when RT-PCR is inaccessible or results are negative, provided there is clinical suspicion. A patient with negative HRCT requires complimentary RT-PCR and possibly follow up CT scan if symptoms persist before treating for COVID-19.
